# Orbital Infection by Saksenaea vasiformis in an Immunocompetent Host

**DOI:** 10.1155/2020/8827074

**Published:** 2020-09-30

**Authors:** Mingkwan Lumyongsatien, Pimkwan Jaru-ampornpan, Mongkol Uiprasertkul, Dinesh Selva

**Affiliations:** ^1^Department of Ophthalmology, Mettapracharak Hospital, Sampran District, 52 moo2 Rhikhing, Sampran, Nakhon Pathom, Thailand 73210; ^2^Department of Ophthalmology, Faculty of Medicine, Siriraj Hospital, Mahidol University, 2 Arun Amarin Road, Siriraj, Bangkoknoi, Bangkok, Thailand 10700; ^3^Department of Pathology, Faculty of Medicine, Siriraj Hospital, Mahidol University, 2 Arun Amarin road, Siriraj, Bangkoknoi, Bangkok, Thailand 10700; ^4^Department of Ophthalmology and Visual Sciences, South Australian Institute of Ophthalmology, University of Adelaide, Adelaide, South Australia, Australia

## Abstract

Orbital mucormycosis caused by *Saksenaea vasiformis* is extremely rare. Herein, we report an immunocompetent 22-year-old Thai female who presented with two months of progressive right upper eyelid mass, associated with swelling, redness, and ptosis. She failed to improve despite multiple courses of antibiotic and steroid treatment. Computed tomography (CT) scan showed infiltration involving the upper eyelid and lacrimal gland. Fungal hyphae were revealed by histopathological study. Polymerase chain reaction (PCR) was positive for *Saksenaea vasiformis* (GenBank: accession number FR687327.1). The patient was successfully treated with surgical debridement, amphotericin B, and oral posaconazole.

## 1. Introduction

*Saksenaea vasiformis* is a fungus of the phylum Zygomycota and order *Mucorales*; it was first isolated from a soil sample in India and reported by Saksena in 1953 [1]. It is a rare zygomycosis typically associated with cutaneous and subcutaneous infection in immunocompetent hosts [2]. However, severe infections such as osteomyelitis, renal infection, rhino-orbito-cerebral infection, and disseminated forms are also documented [3, 4]. Aggressive invasion to surrounding tissues is the most usual representation of *S. vasiformis* mucormycosis.

## 2. Case Presentation

**Figure 1:**
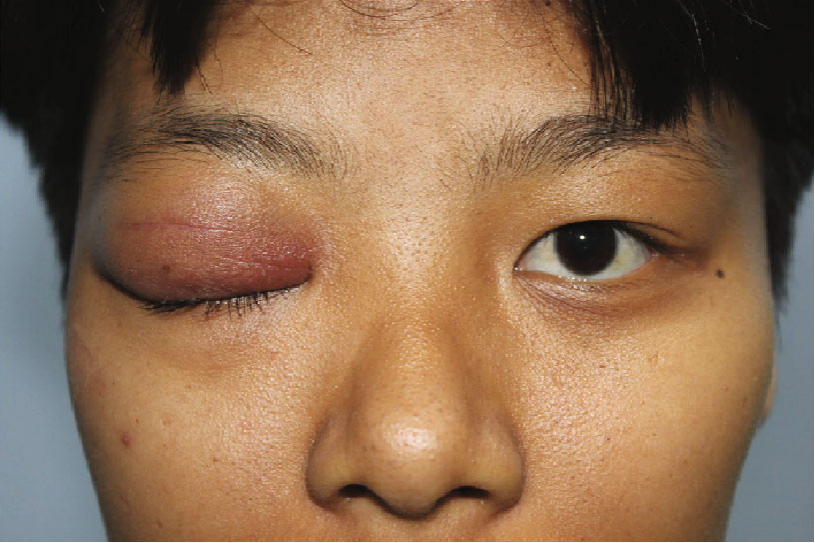
This figure shows the right upper eyelid swelling and ptosis with a palpable firm mass 20 mm in diameter.

**Figure 2:**
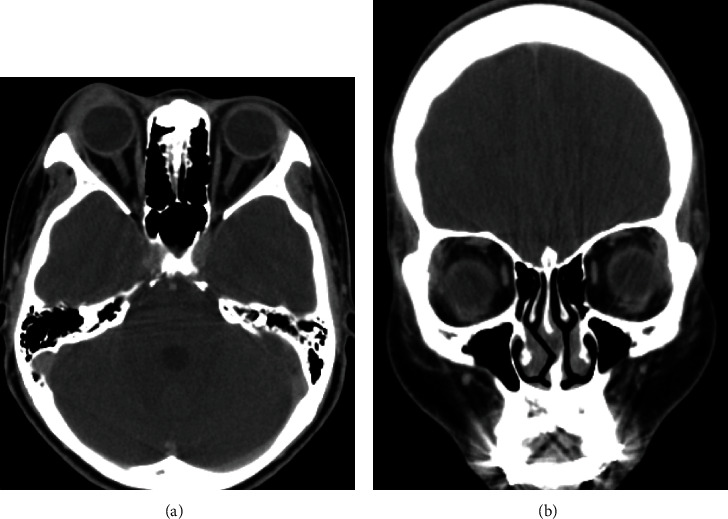
(a) Computed tomography (CT) axial view. (b) Computed tomography (CT) coronal view. (a, b) An infiltrative mass at the right superolateral orbital involving the lacrimal gland and upper eyelid.

**Figure 3:**
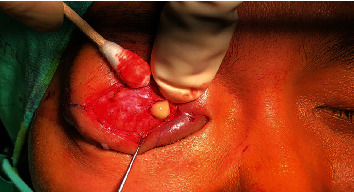
A mass involving the septum and orbital fat with multiple loculated abscesses.

**Figure 4:**
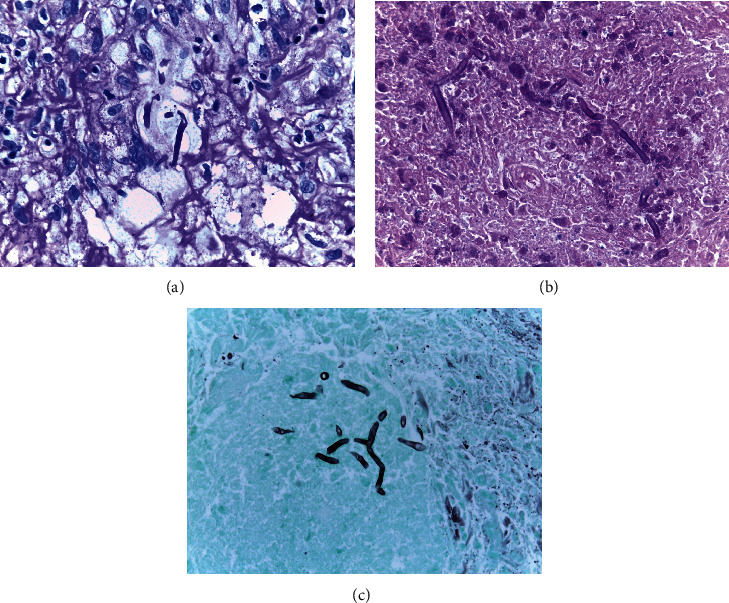
(a) Histopathologic section shows fungal hyphae in granulomatous inflammation background (H&E 400x). (b) Periodic acid-Schiff (PAS) (600x). (c) Grocott methenamine silver (GMS) staining (600x). (b, c) Broad nonseptate and septate hyphae with the right angle branching on PAS and GMS.

**Figure 5:**
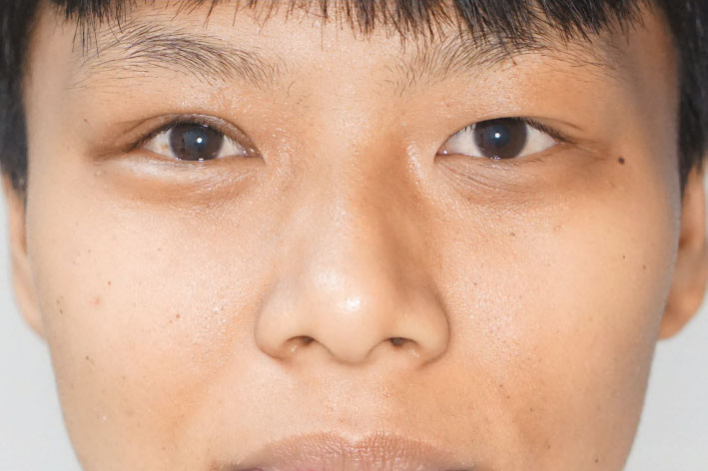
The patient at 3-month follow-up.

A 22-year-old woman with no significant medical history attended a community hospital with gradual swelling, erythematous, and ptosis of the right upper eyelid for two months. The patient had no fever, pain, or visual disturbance. She denied any previous traumatic injury or insect bite. After failure of multiple courses of treatment with dicloxacillin, tetracycline, topical steroid-antibiotic ointment, and oral steroid, she presented to Mettapracharak Eye Center. Physical examination showed the right upper eyelid swelling and ptosis with a nontender palpable firm mass 20 mm in diameter (Figure 1). Best-corrected visual acuities were 20/20 bilaterally. Intraocular pressures were 28 mmHg in the right eye and 16 mmHg in the left eye. Extraocular movements were full, and there was no proptosis. Computed tomography (CT) showed an infiltrative mass at the right superolateral orbital involving the lacrimal gland and upper eyelid (Figures 2(a) and 2(b)). A biopsy was performed via a skin crease incision. A mass involving the septum and orbital fat with multiple loculated abscess was observed intraoperatively (Figure 3). Histopathology demonstrated granulomatous inflammation (Figure 4(a)) with broad nonseptate and sparsely septate hyphae with the right angle branching on periodic acid-Schiff (PAS) (Figure 4(b)) and Grocott methenamine silver (GMS) staining (Figure 4(c)) with no vascular invasion. The patient was commenced on intravenous amphotericin B 40 mg as a 24-hour infusion. Additional surgical debridement was then performed. PCR from the debrided tissue identified *Saksenaea vasiformis* with 98.33% identity (GenBank: accession number FR687327.1). There were negative cultures for bacteria and fungus. There was significant clinical improvement after one week of treatment. However, repeat biopsy on day 28 showed numerous hyphae still present. The patient also developed renal impairment, hypokalemia, and hypomagnesemia attributed to amphotericin B. Hence, amphotericin B was discontinued and replaced by oral posaconazole 300 mg daily for 1 month. Biopsy specimens taken at the end of treatment were negative for fungus. She underwent a levator advancement for residual ptosis. At 3-month follow-up, there was complete resolution of all symptoms and signs (Figure 5).

## 3. Discussion

The most common species of the order Mucorales that relate to mucormycosis in humans are *Rhizopus*, *Mucor*, and *Lichtheimia*. Less than 1% to 5% of the reported cases associate with *Cunninghamella*, *Apophysomyces*, *Saksenaea*, *Cokeromyces*, *Rhizomucor*, *Syncephalastrum*, and *Actinomucor* [4]. *Saksenaea vasiformis* and *Apophysomyces* are usually responsible for infections in immunocompetent hosts usually following trauma with soil contamination. However, direct inoculation via respiratory system has also been reported. The route of entry in our case remains unclear, although unnoticed minor trauma or an insect bite may be a possibility.

*Saksenaea vasiformis* infection was first reported in the facial wounds of a young man following an accident in 1976 [5]. Subsequently, 46 cases have been documented [6]. *S. vasiformis* orbital involvement is extremely rare with 3 published cases associated with sinusitis [3, 7, 8] and 1 individual with isolated orbital infection after aquatic exposure [2]. The prognosis for rhino-orbito-cerebral Saksenaea infection appears to be poor with 83% of mortality rate [3, 4].

Tissue specimens with mucormycosis histopathological identification are often negative cultures. In a review of 929 reported cases of mucormycosis, only 50% were found to be culture positive [9]. Identification of Saksenaea can be a challenge because it fails to sporulate in routine mycological media. Sporulation can be induced in nutrient-depleted media such as distilled water [10]. Funnel-shaped, pigmented sporangia and rhizoids are pathognomonic for *S. vasiformis* [5].

Molecular-based diagnosis such as pan-fungal polymerase chain reaction (PCR) assays enables prompt identification with high sensitivity and specificity. It may be performed on fresh tissue, paraffin-embedded specimens, cerebrospinal fluid (CSF), bronchoalveolar lavage fluid, vitreous fluid, and blood [11]. The molecular identification of fungal species in our case was achieved from fresh tissue by PCR amplification with pan-fungal primers targeted to internal transcribed spacer (ITS) of ribosomal DNA followed by direct sequencing [12]. The whole ITS1-5.8S-ITS2 region primers were synthesized by Bioneer Corporation, Korea. The Basic Local Alignment Search Tool (BLAST) program of GenBank at NCBI website was employed to search for sequence identifier.

Histopathology demonstrates multiple granulomatous inflammation with central necrosis. Fungal hyphae compatible with Mucormycetes in foci of granulomas and vascular invasion may be seen.

The treatment of *S. vasiformis* is aggressive debridement with systemic antifungal drugs. Vascular invasion and tissue necrosis in mucormycosis can lead to poor antifungal penetration. Thus, surgical debridement is critical for thorough eradication of this fungus. The timing and extent of debridement necessary for mucormycosis remains debatable. One retrospective study supported intraoperative frozen sections for margin control of infected tissues [13]. Another report used repeated biopsies to guide the duration of antifungal treatment [14]. In general, amphotericin B (AmB) is the first-line drug, and lipid formulations are significantly less nephrotoxic and provide more safety at higher doses [4, 15]. Posaconazole, a new generation triazole, can be used as an alternative to amphotericin B in the event of the toxicity [2, 4, 5].

## 4. Conclusions

*Saksenaea vasiformis* is an emerging fungal pathogen in humans that often affects immunocompetent hosts. As for the localized infection of periorbital, face, and head areas, sinus-rhino-orbito-cerebral infections are the most common associated sites, followed by cutaneous and subcutaneous infections. The clinical manifestations may be presented as granulomatous or necrotic lesions, cellulitis, or abscesses with purulent exudate and necrotic eschars. The diagnosis relies on a constellation of a high index of suspicion on lesions that fail to respond with conventional treatments or necrotic lesions related to previous trauma. Histopathological examination of the tissues is the gold standard for definite diagnosis of zygomycosis. Molecular-based diagnosis is a practical identification method for Zygomycetes species, so that appropriate treatment can be instigated based on antifungal sensitivities. Aggressive surgical debridement with systemic antifungal drugs is the treatment of choice for the disease.
